# Carbonic anhydrase IX is a pH-stat that sets an acidic tumour extracellular pH in vivo

**DOI:** 10.1038/s41416-018-0216-5

**Published:** 2018-09-12

**Authors:** Shen-Han Lee, Dominick McIntyre, Davina Honess, Alzbeta Hulikova, Jesús Pacheco-Torres, Sebastián Cerdán, Pawel Swietach, Adrian L. Harris, John R. Griffiths

**Affiliations:** 1 Cancer Research UK Cambridge Institute, University of Cambridge, Li Ka Shing Centre, Robinson Way, Cambridge, CB2 0RE UK; 2 Singapore Health Services (SingHealth), Department of General Surgery, Singapore General Hospital, Academia, College Road, Singapore, 169856 Singapore; 3 Department of Physiology, Anatomy & Genetics, University of Oxford, Parks Road, Oxford, OX1 3PT UK; 4 Division of Cancer Imaging Research, The Johns Hopkins University School of Medicine, 720 Rutland Avenue, Traylor 211, Baltimore, MD 21205 USA; 5 Instituto de Investigaciones Biomédicas “Alberto Sols”, c/ Arturo Duperier 4, Madrid, 28029 Spain; 6 Weatherall Institute of Molecular Medicine, University of Oxford, John Radcliffe Hospital, Oxford, OX3 9DS UK

**Keywords:** Cancer microenvironment, Cancer metabolism

## Abstract

**Background:**

Tumour carbonic anhydrase IX (CAIX), a hypoxia-inducible tumour-associated cell surface enzyme, is thought to acidify the tumour microenvironment by hydrating CO_2_ to form protons and bicarbonate, but there is no definitive evidence for this in solid tumours in vivo.

**Methods:**

We used ^1^H magnetic resonance spectroscopic imaging (MRSI) of the extracellular pH probe imidazolyl succinic acid (ISUCA) to measure and spatially map extracellular pH in HCT116 tumours transfected to express CAIX and empty vector controls in SCID mice. We also measured intracellular pH in situ with ^31^P MRS and measured lactate in freeze-clamped tumours.

**Results:**

CAIX-expressing tumours had 0.15 pH-unit lower median extracellular pH than control tumours (pH 6.71 tumour vs pH 6.86 control, *P* = 0.01). Importantly, CAIX expression imposed an upper limit for tumour extracellular pH at 6.93. Despite the increased lactate concentration in CAIX-expressing tumours, ^31^P MRS showed no difference in intracellular pH, suggesting that CAIX acidifies only the tumour extracellular space.

**Conclusions:**

CAIX acidifies the tumour microenvironment, and also provides an extracellular pH control mechanism. We propose that CAIX thus acts as an extracellular pH-stat, maintaining an acidic tumour extracellular pH that is tolerated by cancer cells and favours invasion and metastasis.

## Background

We report the first direct in vivo observation of the isoenzyme carbonic anhydrase IX (CAIX) acting as a pH-stat that sets the extracellular pH (pH_e_) of solid tumours at an acidic level. This is important because the acidic pH_e_ can be tolerated by tumour cells but it inhibits growth of normal host cells, thus facilitating invasion and metastasis.^1,2^

It has long been established that tumour extracellular fluid is acidic^3,4^ but it had not previously been possible to demonstrate a biological pH-stat mechanism by which tumours could stabilise pH_e_. Early discussions about tumour acidification focussed on the lactic acid formed from glucose by aerobic glycolysis (i.e., glycolysis that takes place in tumour cells even at an oxygen concentration high enough to permit oxidative metabolism), a mechanism known as the “Warburg Effect”^5,6^ that is widely regarded as a tumour characteristic.^7^ However, calculations of the output of lactic acid and of CO_2_ from tumours monitored in vivo^8^ suggested that tumour CO_2_ output is comparable to or greater than that of lactate. Human xenografts in nude rats produced 588–850 nmol/g/min CO_2_ vs 527 nmol/g/min lactate (calculated from data in Kallinowski et al.^9^), whereas colonic tumours in human patients produced 1296 nmol/g/min CO_2_ vs 220 nmol/g/min lactate (calculated from data in Holm et al.^10^). Since CO_2_ can be hydrated to form H^+^ and HCO_3_^−^ in the extracellular fluid, in a reaction catalysed by the exofacial (i.e., expressed on the outer surfaces of cells) isoenzyme CAIX, CO_2_ production would tend to acidify pH_e_. The present study has demonstrated this mechanism in vivo.

In recent years, an understanding of why CO_2_ hydration might be important in cancer tissue has begun to emerge from in vitro studies on spheroids composed of CAIX-expressing cancer cells, in which CAIX catalysis reduced pH_e_ and raised intracellular pH (pH_i_).^11,12^ In vivo studies on solid tumours using positron-emission tomography imaging with pH-sensitive low insertion peptides (pHLIP), which are known to bind to cells in more acidic environments, have shown decreased uptake and retention of pHLIP in tumours when CAIX is inhibited by acetazolamide (ATZ).^13^ However, studies attempting to directly measure the effect of CAIX expression on pH_e_ in solid tumours, by using the ^31^P magnetic resonance spectroscopy (MRS) probe 3-aminopropionic acid (3-APP)^13–15^ and hyperpolarized ^13^CO_3_^− 1^H MRS,^15^ have thus far been unable to definitively demonstrate an acidifying effect of CAIX on pH_e_, probably due to technical limits in the resolution and sensitivity of those methods. Hence, causal evidence for CAIX acidifying the tumour microenvironment in vivo has been lacking.

Another long-standing puzzle is whether the acidic pH_e_ of the tumour extracellular fluid is homeostatically controlled at a particular set point and, if so, how that is accomplished. Stubbs et al.^16^ postulated the existence of an extracellular pH-stat as a homeostatic mechanism for pH_e_ that would curtail excessive acid loading, and recent studies have suggested that the ability of cancer cells to modulate the set point of tumour pH_e_ may be an evolutionary strategy to achieve a stable state favouring invasion in response to microenvironmental selection pressures.^17,18^ Several in vitro studies have suggested that the properties of CAIX fit it for a role as an extracellular pH-stat.^12,17,19^ No published study has yet directly demonstrated such a mechanism in vivo, however.

**Fig. 1:**
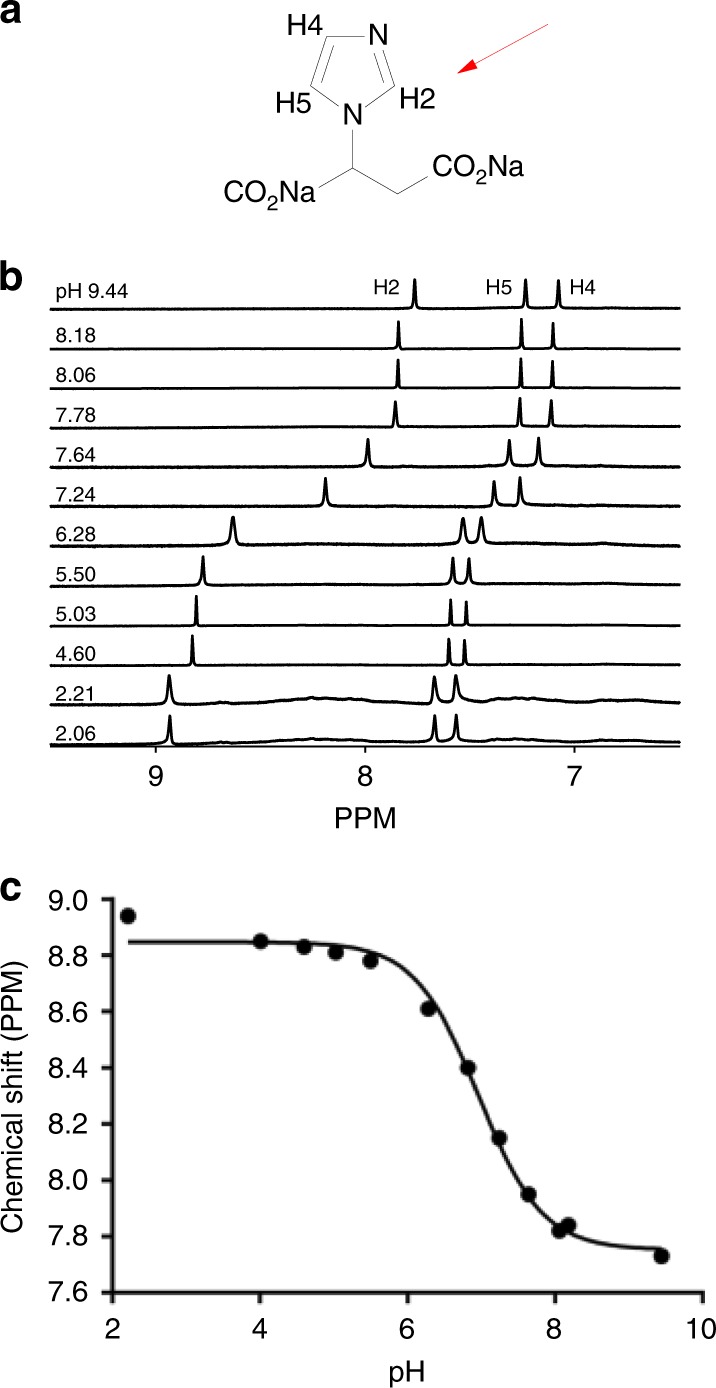
Structural formula and NMR characteristics of the pH_e_ probe ISUCA. **a** Structural formula of ISUCA, indicating the position of the H2 (red arrow), H4 AND H5 nuclei on the imidazole ring. **b** In vitro ^1^H MR spectra of ISUCA at different pH values in murine plasma at 37 °C, showing changes in chemical shift of the H2 peak with changes in pH. **c** pH calibration curve of the chemical shift of the ISUCA H2 peak in plasma at 37 °C, fitted to the Henderson–Hasselbalch equation

We used the probe ISUCA ((±)2-(imidazol-1-yl) succinic acid) which reports tissue pH_e_ from the ratio of its protonated and unprotonated forms, measured from the pH-sensitive chemical shift of its H2 proton (Fig. 1); it has previously been used to map pH_e_ in rat brain tumours.^20^ ISUCA has rapid, carbonic anhydrase (CA)-independent H^+^ equilibration properties, and does not enter the cell, so it reports on pH_e_ with no contaminating signal from pH_i_.^20^ Furthermore, since the ^1^H MRS used with ISUCA is about 15-fold more sensitive than the ^31^P MRS used with 3-APP, it is possible to monitor the pH_e_ of multiple voxels in a tumour (and to discard those with inadequate ISUCA uptake or contamination by non-tumour tissues), whereas 3-APP studies usually obtain only a single reading from a voxel encompassing the whole tumour.

Using ^1^H MRS imaging to detect ISUCA, we were able to measure pH_e_ from multiple voxels in HCT116 colorectal tumour xenografts in mice bearing a tumour model in which all cells expressed CAIX (CA9 tumours) and also size-matched empty vector (EV) tumours that expressed lower levels of CAIX only in hypoxic areas (note that we use CAIX for the carbonic anhydrase IX enzyme, *CA9* for the corresponding gene and CA9 for the HCT116 tumour line that constitutively expresses the enzyme). Hence, the entire extracellular viable volume of CA9 tumours was influenced by expression of CAIX, compared with the relatively small volume in EV tumours where CAIX was induced only at the hypoxic margin.

We observed that CAIX expression in the CA9 tumours imposed an upper limit on tumour pH_e_, thus lowering the steady-state pH in their extracellular space. Furthermore, we demonstrated that CAIX acidifies only the extracellular space, since ^31^P MRS measurements showed no difference in tumour pH_i_ despite increased lactate production. Our findings thus constitute definitive evidence for the long-postulated roles of CAIX in both acidifying the tumour microenvironment and acting as a pH-stat for it.

## Materials and methods

### Cell culture

HCT116 cancer cells transfected with the *CA9* gene coding sequence (HCT116-CA9) or an empty vector (HCT116-EV) were grown in culture as described previously.^12^ The cell lines were provided by Professor A.L. Harris (November 2008) and their identities were verified by short tandem repeat analysis using the PowerPlex 18 Primer Kit (Promega) and their amplicons separated by capillary electrophoresis on AbiPrism genetic analysers. Cells were routinely tested for mycoplasma contamination.

### pH_e_ gradient measurements in 3D cell spheroids

HCT116 cell spheroids (radius 120–130 µm) were cultured using the hanging drop method described previously.^21^ Radial pH_e_ measurements were performed on these spheroids by confocal microscopy (pixel volume of 2 × 2 µm), using a published method.^11^ Measurements were performed in the presence or absence of ISUCA (13.3 mM). The dye was membrane-tethered WGA (wheat germ agglutinin) conjugated fluorescein, excited at 488 nm and emission collected above 520 nm (fluorescence is quenched at low pH).

The average radial pH_e_ gradients were measured in control spheroids (treated with the CA inhibitor ATZ only) and in spheroids incubated with ISUCA (13.3 mM) and ATZ for 2 h (*n* = 29). The diffusion coefficient of ISUCA (D_ISUCA_) was estimated using a published modelling framework.^11^ To investigate the magnitude of pH measurement error introduced by ISUCA, pH_e_ at the core of the spheroid mass was simulated for a model spheroid under different experimental conditions, using a previously published quantitative diffusion-reaction model.^11^

### Tumours

All animal experiments were performed under UK Home Office Licences 80/2203 and 70/7676. Tumours were grown subcutaneously as described previously^12^ but in female severe combined immunodeficient (SCID) mice (CB17/lcr-*Prkdc* ^*scid*^/lcrCrl) obtained from Charles River Laboratories (Margate, UK) at 6 to 8 weeks of age. Tumours were initiated after a minimum of 7 days post-delivery acclimatisation and scanned at a volume of 300–500 mm^3^. Mice were housed in individually ventilated cages, up to 5 per cage, with food and water ad libitum and kept under a light–dark cycle.

### In vitro characterisation of ISUCA

The chemical synthesis of ISUCA and generation of a pH calibration curve using the chemical shift of the ISUCA H2 proton have been previously described.^20^ The spectrum contains three peaks of equal area for the H2, H4 and H5 protons (Fig. 1a); the chemical shift of the H2 peak is most pH sensitive (Fig. 1b), and this peak was used for pH measurement relative to the water resonance, which served as the internal standard. Calibration of pH was confirmed on a 600 MHz Bruker AVANCE NMR Spectrometer (Bruker Biospin, Coventry UK), using solutions of ISUCA in murine plasma at 37 °C with pH values over the range 2.06–9.44 (Fig. 1c).

### Measurement of membrane CA catalytic activity

CA catalytic activity was determined on intact HCT116, EV and CA9 cells in the presence and absence of 100 μM ATZ by means of live-cell fluorescence imaging methods published previously.^11^ In previous studies on the HCT116 line we had shown that its CAIX activity was inhibited by AP105, a CAIX-specific inhibitor,^11^ and that exofacial CA activity in these cells is blocked by a CAIX-specific monoclonal antibody.^22^ The CA-catalysed CO_2_ hydration rate was determined in fractionated cell lysates, using a previously published activity assay.^23^

### In vivo magnetic resonance spectroscopy of tumours

We devised an experimental protocol for the delivery and detection of ISUCA in tumour-bearing mice, followed by automated chemical shift assignment and peak quantitation of the ISUCA H2 peak by ^1^H MRS imaging (MRSI), which permitted direct in vivo measurement and spatial mapping of the voxel-wise distribution of pH_e_. This method allowed us to obtain data from multiple voxels in each tumour and to discard any in which the ISUCA concentration was inadequate or in which skeletal muscle was present in the T2-weighted image.

Our method requires consistent delivery of high ISUCA concentrations into the tumour extracellular space, so timing of ISUCA delivery is crucial for obtaining spectra of adequate quality. For ^1^H MRS of ISUCA to measure pH_e_, anaesthesia was induced by inhalation of 2% isoflurane in oxygen and maintained with 1.0 to 1.5% isoflurane. With the mouse in an appropriate jig, the tumour was positioned centrally in a 20 mm-diameter receive-only surface coil (Rapid Biomedical, Rimpar, Germany). Respiratory rate was monitored using a pneumatic pillow sensor; body temperature, monitored with a rectal probe, was maintained at 37 °C with feedback-controlled warm air delivery (SA Instruments Inc., Stony Brook, NY). The entire assembly was inserted in a ^1^H quadrature volume coil (Rapid Biomedical), positioned inside a horizontal-bore 9.4-Tesla magnet, interfaced to an MRI system scanner console (Agilent, Yarnton, UK) running *VnmrJ 3.1* NMR acquisition and processing software.

T_2_-weighted fast spin echo images were acquired in the axial, coronal and sagittal planes (repetition time (TR) 2000 ms; echo time (TE) 40 ms; field of view (FOV) 30 mm × 30 mm; data matrix 256 × 256; slice thickness 1 mm with 0.5 mm gaps; 5–7 interleaved imaging slices). A volume of interest (VOI) measuring 30 × 30 × 5 mm^3^ was carefully oriented to encompass the tumour in the coronal plane. Localised shimming resulted in a water peak with a line-width of typically 60–80 Hz.

After shimming, a 200 µl bolus of ISUCA solution (1.4 M, pH 7.2) was administered via a pre-inserted peritoneal cannula. An unsuppressed water reference spectroscopic image was then acquired for magnetic field inhomogeneity correction. At 20 min after ISUCA administration, ^1^H MRSI was performed on the VOI to acquire ISUCA spectra from the tumour, using the PRESS-CSI sequence (TR = 1.5 s; TE = 30 ms; FOV 30 mm × 30 mm; 16 × 16 phase encoding steps, 8 averages, sweep width 4032 Hz; slice thickness 5 mm, voxel volume 17.6 µl, acquisition time 52 min). VAPOR (variable power and optimised relaxation delays) water suppression^24^ was employed. Preliminary experiments showed that this timing afforded suitable tumour ISUCA concentrations during the acquisition.

^31^P MRS experiments to measure pH_i_ were carried out in a separate cohort of mice, as the total experimental duration for both studies in the same mouse would have exceeded animal welfare limits. Preparation for the study was similar to that for the ^1^H study, except that the mice were scanned in a 40 mm inside-diameter Millipede volume imaging coil (Agilent) with a custom-made three-dimensional (3D)-printed insert containing a 25 mm ^31^P surface coil, which enclosed the tumour. ISIS (image-selected in vivo spectroscopy) localisation was used with 8 ms hyperbolic secant adiabatic inversion pulses and 5 ms BIR-4 adiabatic excitation pulses. Outer volume suppression was employed to minimise contamination from adjacent tissues. The median voxel volume in these single-voxel studies was 250 µl. No exogenous agent was injected, as the pH_i_ was calculated from the chemical shift of the inorganic phosphate (Pi) peak, which varies with pH in the physiological range and is predominantly detected from the intracellular volume.^25^ See Supplementary Methods for details of spectroscopic data processing. Tumour lactate was assayed colourimetrically, see Supplementary Methods.

After data acquisition, the mouse was immediately killed and the tumour excised and fixed.

### Histopathology

Tumours were excised immediately after pH spectroscopy, fixed for 24 h in 10% formalin and embedded into paraffin blocks (Leica ASP300S automated processor). The 3 µm sections were cut and where possible adjacent sections were stained with haematoxylin and eosin and for CAIX and Ki67. Automated immunohistochemistry was performed on a Leica BOND-MAX system. The CAIX antibody (monoclonal mouse anti-human, M75 clone) was a gift from Dr. Pastorek, Bratislava, Slovakia, and was used in a 1:100 dilution with pH 6.0 citrate buffer antigen retrieval; the Ki67 antibody (monoclonal mouse anti-human, Dako M7240) was used in a 1:200 dilution with pH 9.0 Tris-EDTA antigen retrieval. DAB enhancer (Leica 9432) was used for both antibodies. Slides were scanned using the Aperio (UK) ScanScope system at ×20 magnification.

### Lactate analysis

Tumours (CA9 constitutive expressers and EV5 empty vectors) were excised from terminally anesthetised mice and immediately freeze-clamped using tongs previously immersed in liquid nitrogen. Samples were powdered in liquid nitrogen and extracted with 4 volumes of 6% perchloric acid followed by centrifugation and neutralisation. Lactate was assayed in the extracts using the L-Lactate Assay Kit (Colorimetric) ab65331 (Abcam) according to the manufacturer’s instructions. Since lactate is a passively distributed solute, total tissue measurements cannot attribute its location relative to the cell membrane. The pH distribution was therefore used to infer the intracellular to extracellular lactate gradient.

### Statistics

Statistical analyses were performed using GraphPad Prism software, Version 5.01 (GraphPad Software, Inc., La Jolla, CA, USA). For pH or rate constant data, differences between means were tested by a two-tailed *t*-test. For animal experiments, statistical differences were tested with the non-parametric Mann–Whitney test, while correlations were tested with the non-parametric Spearman’s rank correlation coefficient. For the in vitro studies, the data are presented as the means ± standard errors and were tested for statistical differences with a two-tailed *t*-test. Differences were considered statistically significant if *P* < 0.05.

## Results

**Fig. 2:**
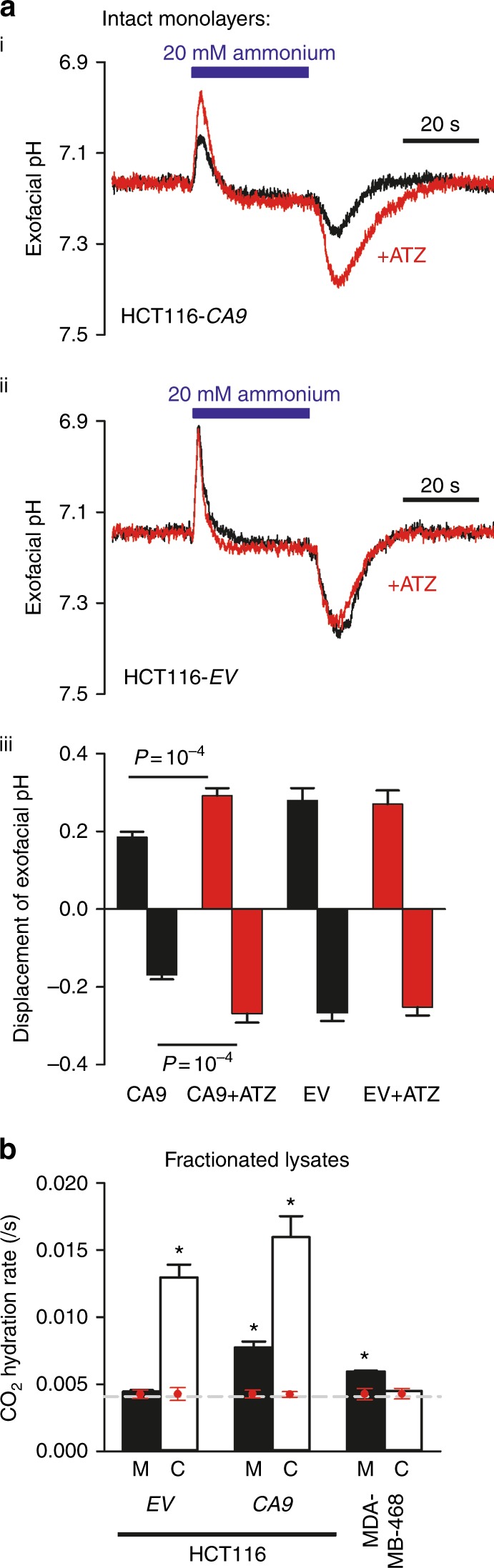
Exofacial CA activity in HCT116 CA9 cells is in the physiological range of activity. **a** Exofacial pH transients measured with surface membrane-tagging WGA fluorescein in response to a 30 s pulse of 30 mM NH_3_/NH_4_^+^-containing superfusate, measured in (i) intact HCT116 CA9 cells (*n* = 15) and (ii) intact HCT116 EV cells (*n* = 9), compared with paired runs in the presence of 100 µM ATZ, a broad-spectrum inhibitor of CA activity. (iii) Mean data for the peak and nadir of pH_e_ transients in CA9 and EV cells, compared to the responses during ATZ inhibition. pH changes were smaller (i.e., better buffered) in CA9 cells, indicating membrane-bound CA activity. **b** CO_2_ hydration rates, normalised to protein concentration, measured in the cytoplasmic (C) and membrane (M) fractions of EV or CA9 HCT116 cells, compared to wild-type MDA-MB-468 cells, a cell line that naturally expresses CAIX in normoxia. Measurements performed on triplicate of independently harvested monolayers. Dashed line indicates spontaneous activity measured in buffer only (no cellular material). Red data points show values for each cell line after inhibition with 100 µM ATZ, showing reduction to spontaneous levels. Error bars are SEM. *indicates p<0.05 for the difference between C and M fractions and spontaneous activity in buffer alone (which is shown by the dashed line)

Using fluorescent dye measurements of cell surface pH transients in response to a 30 s pulse of NH_3_/NH_4_^+^-containing buffer, we verified the presence of exofacial carbonic anhydrase activity in CA9 cells (Fig. 2ai) and its absence in EV cells in vitro (Fig. 2aii). ATZ (100 µM) was added to block CA activity and interrogate pH transients in the absence of CA catalysis. NH_3_/NH_4_^+^-evoked pH_e_ transients were significantly smaller (i.e., better buffered) in CA9 cells (Fig. 2aiii). Note that ATZ is membrane permeable, but inhibition of intracellular CAs cannot meaningfully affect extracellular pH dynamics; therefore, its inhibitory effect is attributable only to exofacial catalytic sites. Using a functional assay for CA activity, CA9 cell membrane fragments were shown to significantly accelerate CO_2_ hydration rate, whereas EV membranes showed no catalytic activity (Fig. 2b). The exofacial CA activity in CA9 cells was comparable to endogenous levels of membrane CA activity in wild-type MDA-MB-468 breast cancer cells, a cancer cell line known to constitutively express CAIX (Fig. 2b), which confirms that CAIX-transfected HCT116 cells have exofacial CA activity in the physiological range.

**Fig. 3:**
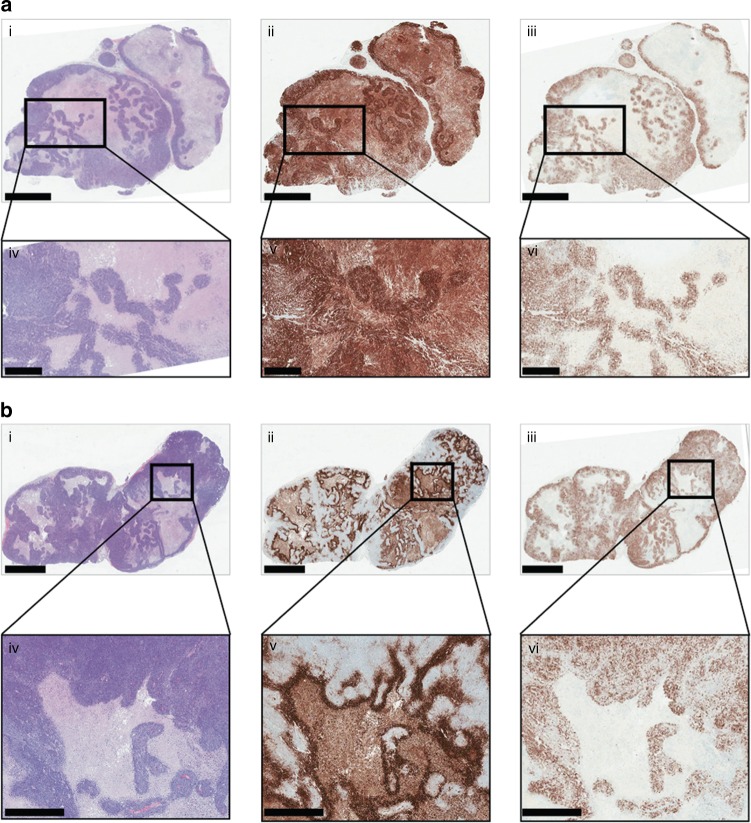
CAIX is expressed in all viable CA9 tumour tissue (**a**) but only at the hypoxic necrotic/viable interface in EV tumours (**b**). For both (**a**) and **b**: upper parts i, ii and iii show close-cut whole tumour sections, scale bar: 3 mm; lower parts iv, v and vi show higher magnification of the area indicated by the black rectangle, scale bar: 800 µm. Parts i and iv: haematoxylin and eosin staining; viable areas are dark purple; necrotic areas are pale. Parts ii and v: CAIX staining in brown, showing staining in all viable areas in CA9 tumours but absence of staining in the viable areas of EV tumours. Parts iii and vi: Ki67 staining (for proliferating cells) in brown, which confirms the proliferative areas and further defines the hypoxic periphery of viable tissue, adjacent to necrosis. Necrotic areas are non-perfused and so do not give an ISUCA signal

**Fig. 4:**
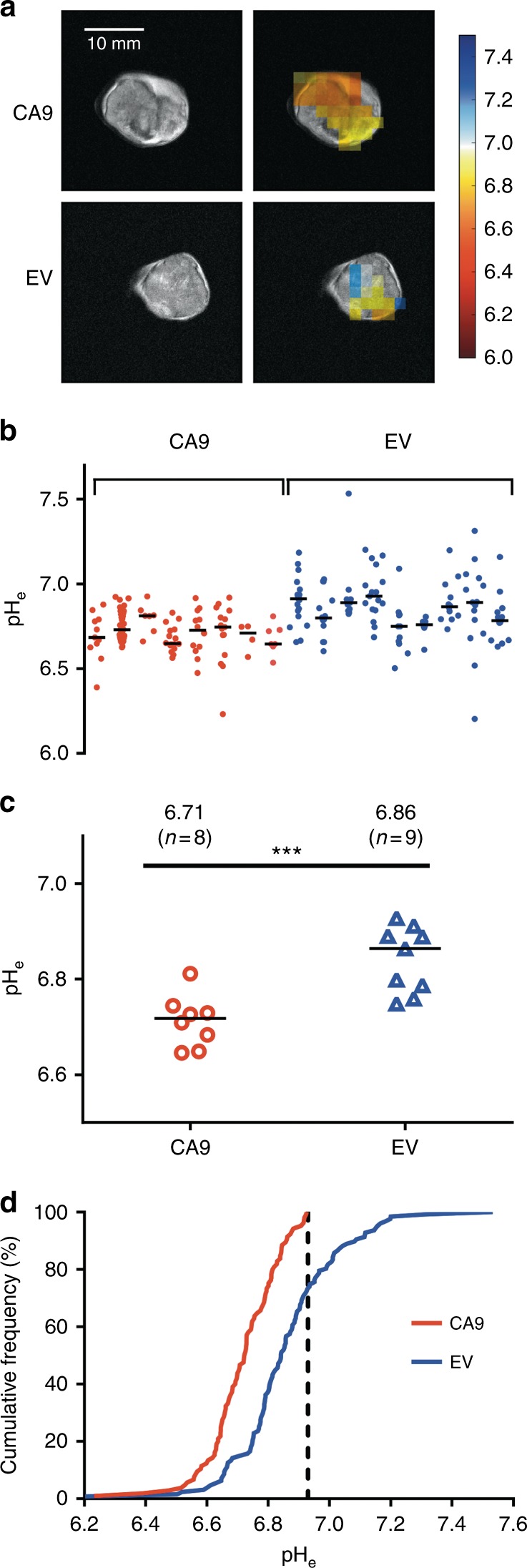
^1^H MRSI of tumour pH_e_ reveals in vivo extracellular acidification by CAIX. **a** False coloured pH_e_ maps of representative CA9 and EV tumours overlaid onto MR images encompassing the MRSI volume of interest, Scale bar: 10 mm. **b** pH values from the pH_e_ maps of each tumour, with the medians. **c** Median tumour pH_e_ values were lower for CA9 tumours (6.71, *n* = 8) than for EV tumours (6.86, *n* = 9; ***P = 0.001, Mann–Whitney test). **d** Cumulative histogram depicting pH_e_ frequency distribution. CA9 tumours did not show any pH_e_ value above 6.93, whereas 26.8% of the values for the EV tumours were above that.

EV tumours expressed CAIX only in cells around hypoxic regions, whereas CAIX was uniformly expressed by all viable CA9 tumour cells (Fig. 3), as had been found by McIntyre et al.^12^ We mapped the pH_e_ of CA9 and EV tumours in vivo by ^1^H MRSI of the ^1^H MRS probe ISUCA. Mapping 5 mm-thick spectroscopic images of in vivo pH_e_ onto MRI images of CA9 and EV tumours (Fig. 4a; Supplementary Figures 1 and 2) revealed a median pH_e_ of 6.71 in the CA9 tumours (*n* = 8), which was significantly more acidic (*P* = 0.001, Mann–Whitney test) than the median pH_e_ of 6.86 in the EV tumours (*n* = 9) (Fig. 4b, c). The range of pH_e_ values in CA9 tumours was 6.23 to 6.93, whereas the range of pH_e_ values in EV tumours was 6.20 to 7.53. Intriguingly, we observed a striking absence of any pH_e_ value above pH 6.93 in the CA9 tumours, whereas 27% of the EV tumour pH_e_ values were above that cut-off value (Fig. 4d). These results suggest that CAIX expression by HCT116 cells imposes an upper pH_e_ limit in solid tumours, akin to the action of a pH-stat.

To confirm our conclusions, we investigated whether the presence of ISUCA in the extracellular fluid affects the accuracy of tumour pH_e_ measurements. ISUCA was capable of buffering absolute changes in pH in cell spheroids but only slightly diminished the size of the pH_e_ gradients (Supplementary Figure 3A, B) The average ISUCA concentration in tumours (13.3 mM, Supplementary Figure 4A) only accelerated CAIX activity in spheroids by 12% (Supplementary Figure 3C), possibly because this imidazole derivative may augment the transfer of H^+^ ions between the bulk solution and the catalytic site.^26^ Despite these minor effects, the lack of positive correlation between ISUCA concentration and tumour pH_e_ suggests that ISUCA is unlikely to have a significant effect on the magnitude of facilitated CO_2_ diffusion in vivo (Supplementary Figure 4B, C and D). In silico modelling of pH_e_ dynamics at the core of spheroids suggests that the presence of ISUCA may lead to a minor underestimation of extracellular acidification (Supplementary Figure 5). Collectively, these lines of evidence suggest that the increased acidification observed in CA9 tumours is unlikely to be due to artefacts induced by the chemical buffering properties of ISUCA. Furthermore, even if one of these potential artefacts were to have an unexpectedly large effect, it would only induce spurious results if higher concentrations of ISUCA were present in one of the tumour types, whereas we found that there was no significant difference in these concentrations (Supplementary Figure 4A).

**Fig. 5:**
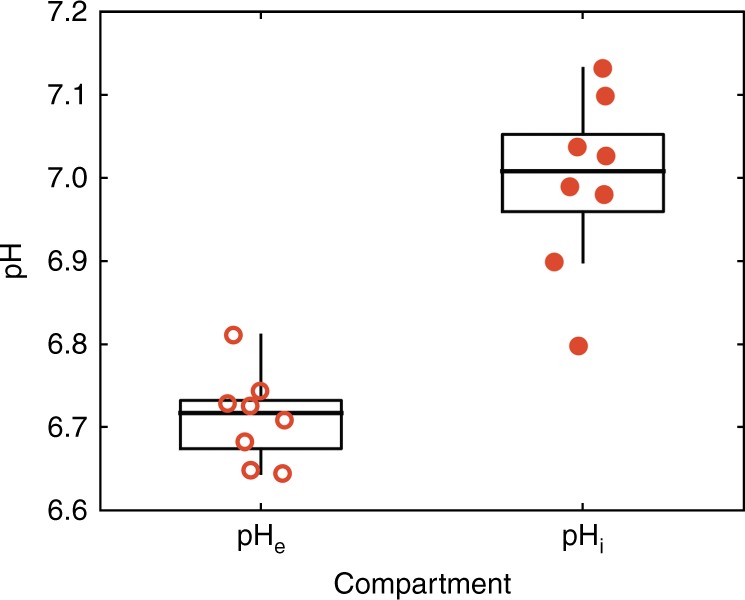
Comparison of in vivo pH_i_ and pH_e_ values for CA9 tumours. Median pH_e_ values were derived from ^1^H MRS of ISUCA and pHe values from ^31^P MRS of endogenous metabolites in a separate cohort. The minimal overlap of the values supports the purely extracellular distribution for ISUCA previously demonstrated in vitro with C6 cells by Provent et al.^20^

**Fig. 6:**
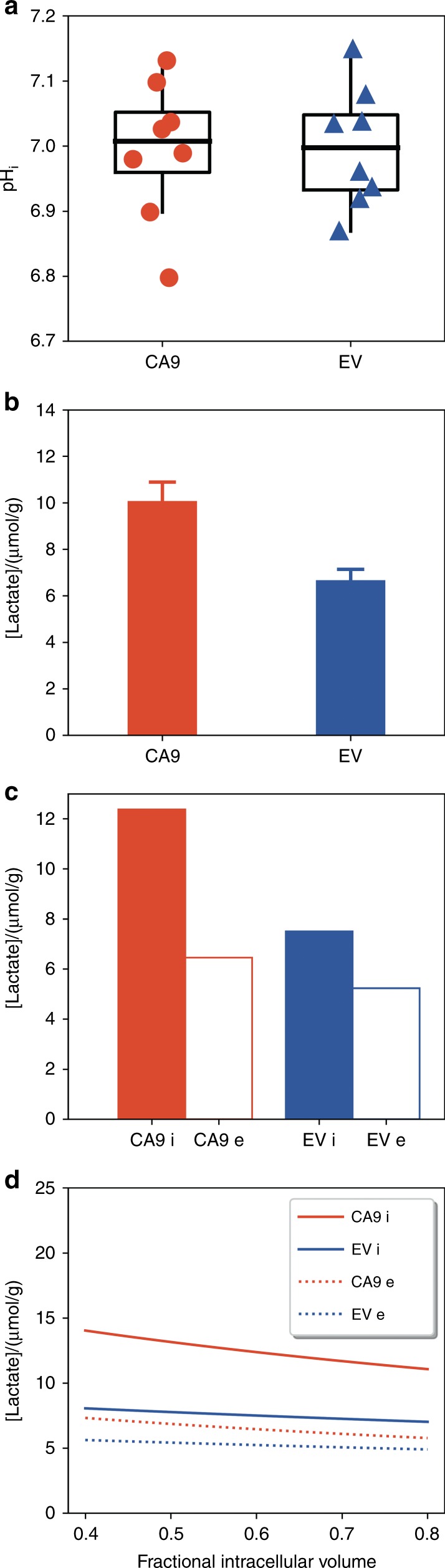
CAIX-expressing HCT116 tumours do not show increased alkalinisation in vivo and have a higher lactate retention. **a** ^31^P MRS measurements of pH in 8 CA9 and 8 EV5 tumours. The pH_i_ of CA9 tumours was 7.00 ± 0.04 (mean ± SEM) and of EV tumours was 6.99 ± 0.04 (*P* = ns). This data group, with a pooled error variance of 0.010, would be powered at 0.88 for detection of the hypothesized 0.15-unit alkalinisation using a one-sided *t*-test. **b** Total lactate levels measured in excised tumours. **c** Calculated intracellular and extracellular lactate concentrations, assuming an intracellular fraction of 0.6; note that there are no error bars as the calculation was based on the mean lactate levels. **d** Variation of calculated intracellular and extracellular lactate concentrations with intracellular fraction

To investigate whether extracellular acidification was accompanied by intracellular alkalinisation in vivo, we obtained the pH_i_ of CA9 and EV tumours from ^31^P MRS measurements of the endogenous inorganic phosphate signal, the chemical shift of which is pH sensitive in the physiological range and which predominantly arises from the intracellular compartment of tumours.^3^ The ranges of pH_e_ and pH_i_ values in the CA9 tumours (Fig. 5) show minimal overlap, demonstrating that markedly differing extracellular and intracellular pH environments were probed, and we found no detectable differences between the pH_i_ values of the CA9 tumours and the EV tumours (7.00 ± 0.04 vs 6.99 ± 0.04; mean ± SEM; Fig. 6a).

To confirm the validity of our pH imaging results, we performed a biochemical assay for lactate to test whether there are differences in lactate retention between CA9 and EV tumours. Lactate ions are known to distribute passively across the cell membrane, both in tumour cells^27^ and solid tumours,^28^ transported by MCT proteins,^29^ with the intracellular to extracellular lactate concentration ratio depending on the transmembrane pH gradient. Cells bathed in a more acidic milieu, such as CA9 tumour cells, are therefore expected to retain more lactate. We measured total lactate concentrations in freeze-clamped CA9 and EV tumours (see Supplementary Methods). The CA9 tumours had significantly higher total lactate content than the EV tumours (10.01 ± 0.55 μmol/g, *n* = 10 vs 6.6 ± 0.89 µmol/g, *n* = 6; *P* = 0.01, see Fig. 6b). Since the mean pH_i_ and pH_e_ for each tumour type are known, we can therefore calculate the ratio of lactate on either side of the membrane. Using the mean values from Fig. 6b we calculate the intracellular and extracellular lactate values shown in Fig. 6c: the CA9 tumours had markedly higher intracellular lactate than the EV tumours, whereas the difference in extracellular lactate was much less. This calculation assumed an intracellular volume fraction of 0.6, but a sensitivity analysis (Fig. 6d) shows qualitatively similar results across the full reasonable range of intracellular volume fractions (0.4–0.8); for comparison, Panagiotaki et al.^30^ reported intracellular volume fractions of 0.84 ± 0.02 and 0.68 ± 0.02 in two other human colorectal xenograft models.

In conclusion, we demonstrated (i) that exofacial CAIX expression by HCT116 tumours resulted in reduced tumour pH_e_; (ii) that the maximum pH_e_ of their extracellular space was held below 6.93; (iii) that their pHi was unaffected; and (iv) that they would therefore retain intracellular lactate.

## Discussion

The HCT116 CA9 colorectal tumours uniformly expressing high levels of CAIX protein, along with their EV counterparts in which CAIX is induced only in hypoxic regions, offer a tractable experimental platform to study the effect of CAIX expression on tumour pH_e_ in vivo, independent of changes in tumour vascularity, oxygenation and metabolism. The CA9 tumours present an adequate volume of viable tissue in which pH_e_ is influenced by CAIX to allow accurate pH_e_ measurements that can be compared with similar volumes of EV tissue which have much less CAIX expression. Using the ISUCA method, we have obtained multiple pH_e_ values from each tumour and discarded voxels with inadequate ISUCA uptake or skeletal muscle contamination. Our results thus provide strong evidence that exofacial expression of CAIX on cancer cells results in a lower pH_e_. We have also shown that exofacial CAIX catalysis acts in vivo as a pH-stat mechanism that tends to maintain a mildly acidic pH_e_. We infer that this will occur not only within regions of solid tumours in which CAIX is expressed because of hypoxia, but also in any other area where CAIX is expressed independently of hypoxia. While CAIX is usually associated with tumour hypoxia and frequently used as a hypoxia marker, it is expressed in normoxia in the oestrogen receptor-positive MCF7 cell line^31^ and multiple studies have shown that it is often expressed in vivo in areas where hypoxia is not detected.^32–34^

In previous studies on CAIX expression in HCT116 spheroids, the extracellular acidification was accompanied by an intracellular alkalinisation.^11^ We therefore hypothesised that if extracellular acidification by CAIX activity was due to a transmembrane redistribution of acidity, the intracellular compartment of CA9 tumours would be more alkaline than that of the EV tumours. The absence of detectable differences between the pH_i_ values of the CA9 tumours and the EV tumours (7.00 ± 0.04 vs 6.99 ± 0.04; mean ± SEM) (Fig. 6a) suggests that exofacial CAIX expression has little net effect on tumour pH_i_ in vivo and that the effect of CAIX expression is primarily on tumour pH_e._ This might be due to the weaker buffering capacity and smaller volume of the extracellular space compared with the intracellular space, both of which would tend to amplify acid-load induced pH_e_ changes. The calculated intracellular and extracellular lactate concentrations (Fig. 6c) predict that the more positive pH_i_–pH_e_ gradient in CA9 tumours would retain lactate intracellularly at the steady state, and this was demonstrated by measuring lactate in excised tumours (Fig. 6b) and calculating intra- and extracellular lactate (Fig. 6c). Exofacial carbonic anhydrases (mostly CAIX) facilitate CO_2_ diffusion across extracellular spaces, and by doing so acidify the extracellular space en route. The fall in pH_e_ reduces the thermodynamic driving force for removing lactate from cells and therefore raises intracellular lactate. Notwithstanding the different methods for assay, the minimal overlap in the pH_e_ and pH_i_ values of the CA9 tumours (Fig. 5) also demonstrates that ISUCA reports pH_e_ in vivo, substantiating the previous in vitro report in cultured C6 cells.^20^

The acidic pH_e_ of solid tumours has been hypothesised to advantage cancer cell growth by killing adjacent normal cells, promoting extracellular matrix degradation and thus remodelling normal tissue architecture and modulating various steps along the invasion-metastasis cascade.^1,2^ Importantly, recent studies have uncovered evidence for increased mitochondrial oxidative metabolism (and thus CO_2_ production) in migratory/invasive cancer cells^35^ and also in vivo in a variety of preclinical tumour models^36,37^ and human cancers.^38^ Estrella et al.,^39^ using a window-chamber model, confirmed that tumours invaded the normal tissue that was most acidic, at pH_e_ values below a threshold that ranged from 7.1 to 6.8 in individual mice (their Fig. 2b, d). In our study 44% of the measured CA9 tumour pH_e_ values were below 6.7, whereas the EV tumours had only 15% (Fig. 3), showing that CA9 tumours produced sufficient CAIX-related acidification in vivo to favour invasion and/or metastatic cell escape. This notion is supported by reports of reduction in the incidence of metastases in breast cancer models by CAIX inhibition^40–42^ and in a prostate cancer model by CAIX silencing.^43^ Thus, the pH-stat function of CAIX is likely to extend to the growing margin of the solid tumour, distant from optimal oxygen supplies, allowing leading-edge cells to set tissue pH_e_ at a level inhibitory to normal tissue and thus facilitating invasion of cancer cells. CAIX is also known to be expressed in the lamellipodia at the leading edge of motile normal cells and has been postulated to acidify the adjacent extracellular fluid,^44^ a mechanism that might facilitate the invasion of motile cancer cells into the surrounding normal tissue. Perhaps cancer cells induce extracellular acidification by co-opting a method used by normal cells to facilitate tissue remodelling.

The idea that there should be a homeostatic mechanism that maintains pH_e_ within a physiologically appropriate range was hypothesised by Stubbs et al.^16^ Recent publications have suggested that CAIX could be acting as both the H^+^ ion generator and the pH sensor in such a mechanism. Studies in vitro have found CAIX to be active even at relatively acidic levels, with a pK of 6.49^19^ or 6.81–6.86^12^ and others have suggested that CAIX has its highest catalytic activity at pH 6.8^17^. Moreover, compared to other CA isoforms, CAIX appears to be more sensitive to pH changes near the pK, with a Hill cooperativity number of ~2; these properties suit it ideally to the role of an acidic pH-stat.^12,17^ In contrast, CAXII (another exofacial cancer-related isoform) is active across the pH range and would not act as a pH-stat, since the persistence of CAXII activity at low pH_e_, means that the reaction will continue unopposed.^45^

This study constitutes the first evidence from solid tumours in vivo for the presence of a pH-stat mechanism, which, when supplied with CO_2_, would tend to stabilise an acidic pH_e_ within CAIX-expressing regions of tumours. We propose, therefore, that whereas other membrane H^+^ transport mechanisms such as the Na^+^/H^+^-exchanger and the vacuolar ATP-dependent H^+^ pump are primarily concerned with maintaining a constant pH_i_, extracellular CA catalysis endows CAIX-expressing cancer cells with the ability to set the pH of their environment at an acidic level. We suggest that this pH-stat mechanism may be found to operate in other human cancers, and perhaps in other disease states involving carbonic anhydrases, tissue ischaemia and pH changes, such as ischaemic cardiomyopathy^46^ and diabetic retinopathy.^47^

Our findings support the involvement of CAIX in determining tissue pH_e_, independently of hypoxia status, and add to the therapeutic rationale for pharmacologically inhibiting CAIX, which is already an emerging drug target.^48^ Abrogation of this pH-stat function might reduce the acidity of the tumour microenvironment and impinge on the downstream disease mechanisms governed by this pathophysiologic feature of cancer.

## Electronic supplementary material

Supplementary Figures

Supplementary Methods

## Availability of data and material

Additional data related to this publication, with associated DOIs, will be made available at the data repositories of the Universities of Oxford and Cambridge.
