# Supplementary material for: Carbonic anhydrase IX is a pH-stat that sets an acidic tumour extracellular pH in vivo
Source: Br J Cancer. 2018 Sep 12;119(5):622–30. doi: 10.1038/s41416-018-0216-5 (PMC6162214; doi:10.1038/s41416-018-0216-5)
Supplement: Supplementary file 1 — Supplementary Figures [file 41416_2018_216_MOESM1_ESM.docx]

**SUPPLEMENTARY FIGURES**


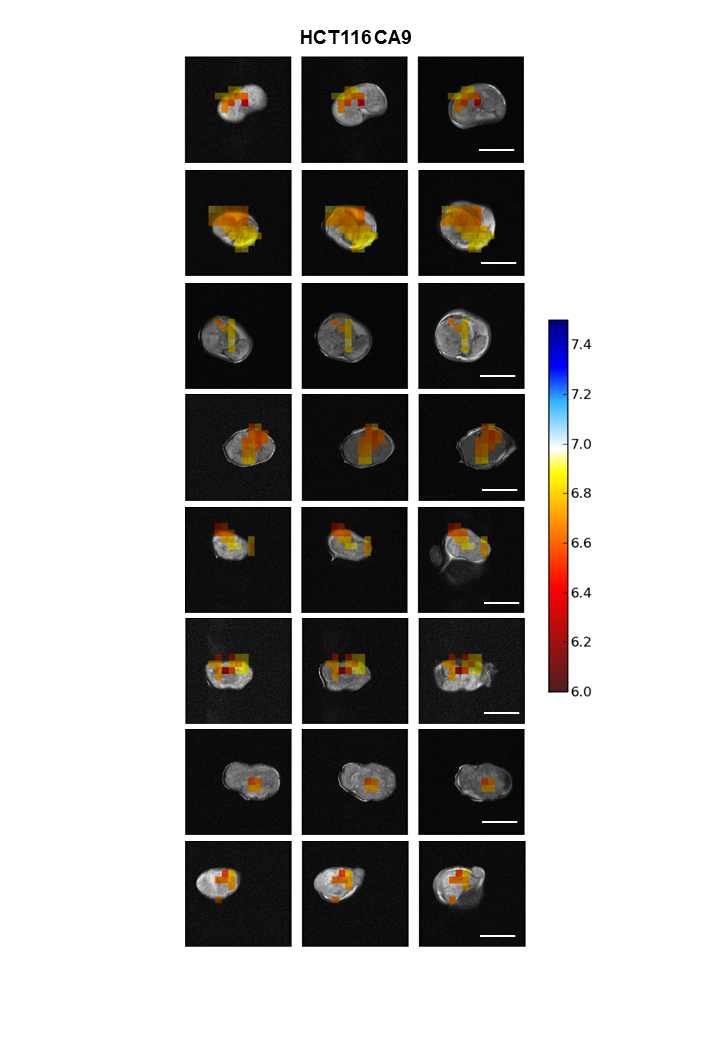


**Supplementary Figure** **1: pH_e_ maps of HCT116CA9 tumours** **(n = 8) overlaid onto coronal T_2_-weighted MR images encompassing the MRSI volume of interest.** The MRSI data were acquired from a 5mm slice, while the MRI slices were spaced 1.5mm apart. Each row shows the MRSI false-colour map for a single tumour overlaid on the coronal images which intersect the full thickness of the MRSI slice, arranged dorsal-to-ventral from left to right. Scale bar: 10 mm.


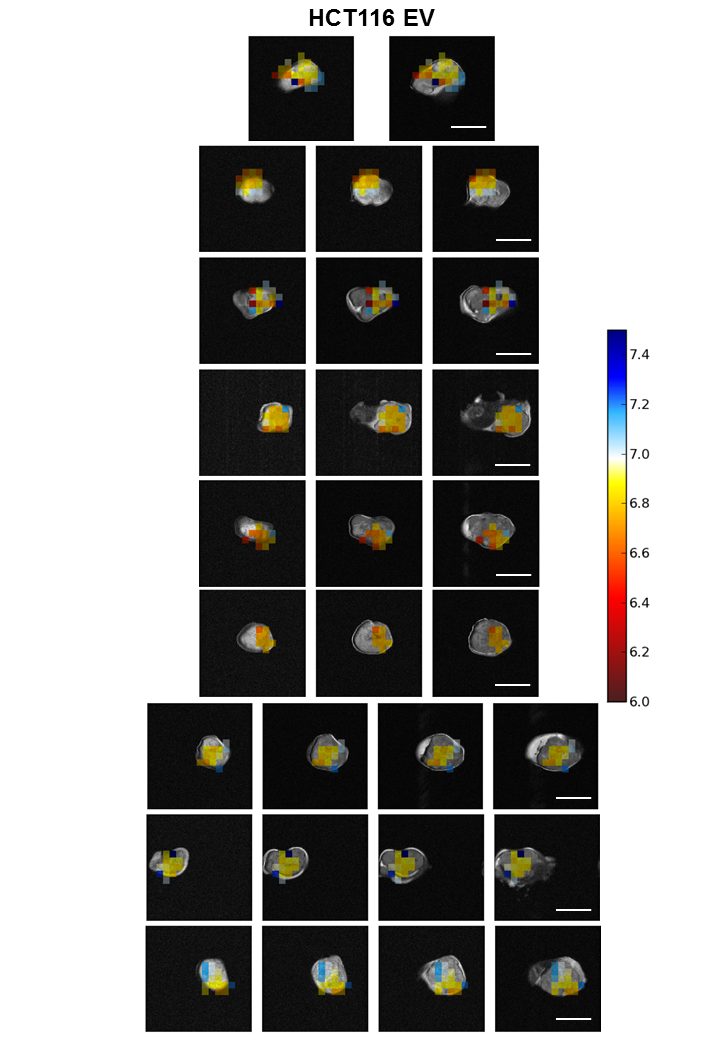


**Supplementary Figure** **2: pH_e_ maps of HCT116EV tumours (n = 9) overlaid onto coronal T_2_-weighted MR images encompassing the MRSI volume of interest.** The set of images in each row was acquired from neighbouring slices in the dorsal-to-ventral direction (from left to right) of the same tumour. Scale bar: 10 mm.


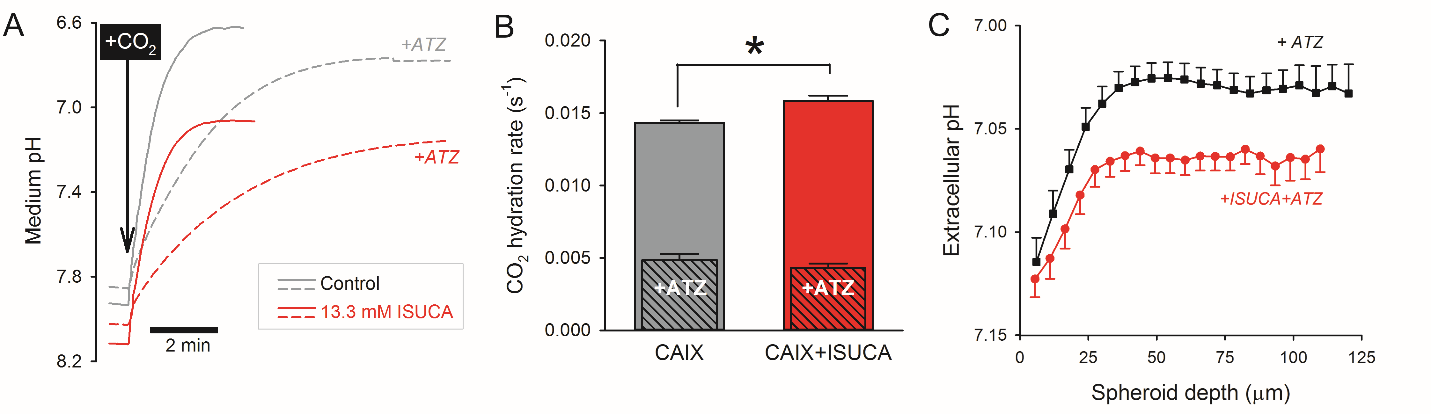


**Supplementary Figure** **3: The effect of ISUCA on tumour pH_e_ measurements is small.** (A) Time-course of medium acidification following injection of CO_2_-saturated water to a buffered suspension of CAIX-containing membrane fragments. The absolute pH change was smaller in the presence of ISUCA, because of its pH buffering properties. Acetazolamide (ATZ) reduced the rate of hydration to spontaneous levels. (B) Best-fitting of data for the CO_2_ hydration constant. CAIX activity was modestly accelerated in the presence of ISUCA (* P<0.05). (C) Radial pH_e_ gradients measured as distance from the core-to-periphery of spheroid (spheroid depth) in the presence and absence of 13.3 mM ISUCA. ISUCA reduced pH_e_ gradients by 32%.

**
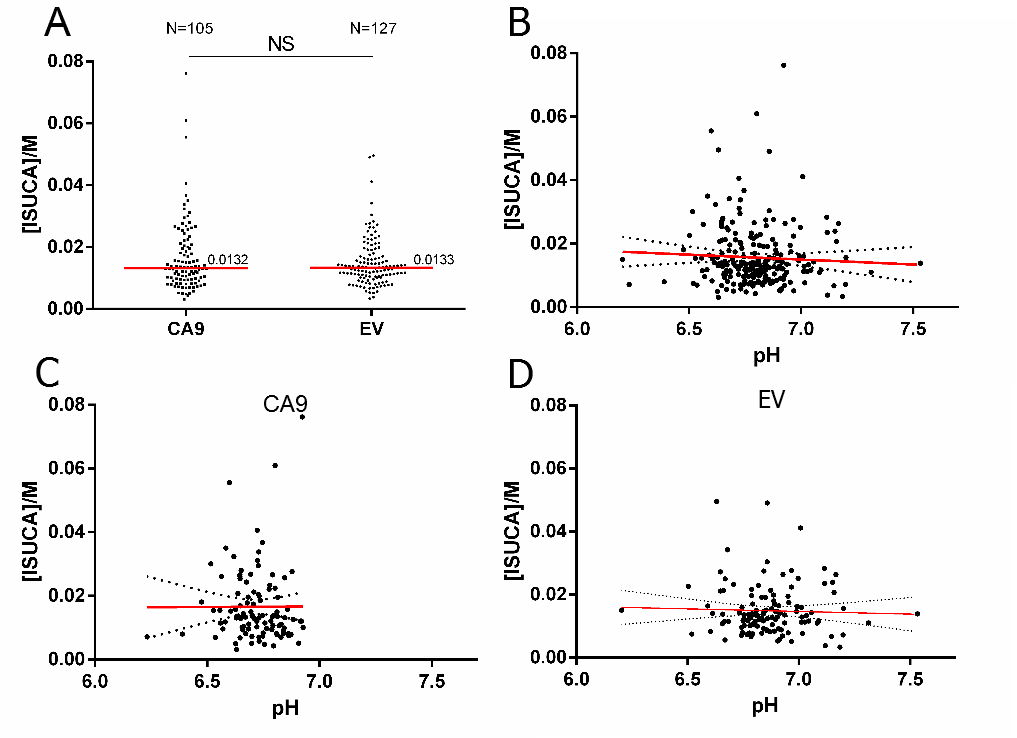
**

**Supplementary Figure** **4: ISUCA concentration in the HCT116 tumours and its correlation with measured pH_e_.** (A) Pooled individual voxel concentrations of ISUCA in the HCT116 tumours. (HCT116 CA9, N=105 voxels from 8 tumours; HCT116 EV, N=127 voxels from 9 tumours).Median ISUCA concentration was not significantly different between the CA9 and EV groups (13.2 mM vs 13.3 mM, respectively; Kruskal-Wallis test). (B) Scatter plot of the ISUCA concentration within each voxel and the voxel’s corresponding measured pH_e_ for the HCT116 tumours (r = -0.083, P=0.21, N=232). (C) Scatter plots of these two variables for the HCT116 CA9 tumours (r = -0.15 P=0.13, N=105). (D) Scatter plot of these two variables for the HCT116 EV tumours (r = 0.0087, P=0.92, N=127. For all analyses (B, C & D), the Spearman’s correlation coefficient showed a lack of correlation between these two variables, and the slopes of the linear regression lines (red) for the two parameters were not significantly different from zero.


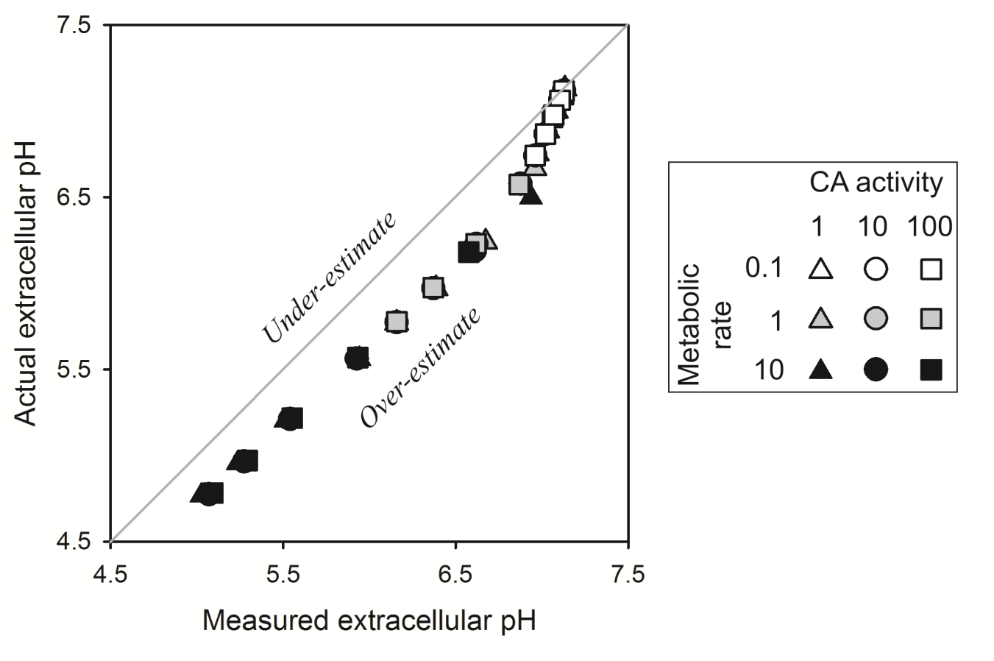


**Supplementary Figure** **5:** ***In Silico*** **Reaction-Diffusion Modelling of pH_e_ at the core of spheroids to estimate the effect of ISUCA on pH_e_ measurements.** Spheroid radius was varied from 100 μm to 500 μm. Extracellular CA activity was varied up to 100-fold the spontaneous rate. Metabolic CO_2_ production ranged from 0.1-fold to 10-fold of the rate described for HCT116 spheroids11.Core-pH_e_ simulated in the presence of 13.3 mM ISUCA (x-axis) was plotted against core-pH_e_ simulated in the absence of ISUCA (y-axis). The difference between the two values provides an estimate of error introduced to pH_e_ measurements by ISUCA.

.
