# Supplementary material for: Carbonic anhydrase IX is a pH-stat that sets an acidic tumour extracellular pH in vivo
Source: Br J Cancer. 2018 Sep 12;119(5):622–30. doi: 10.1038/s41416-018-0216-5 (PMC6162214; doi:10.1038/s41416-018-0216-5)
Supplement: Supplementary file 2 — Supplementary Methods [file 41416_2018_216_MOESM2_ESM.docx]

**SUPPLEMENTARY METHODS**

**^1^H Spectroscopic Imaging Data Processing**

All MRSI datasets were processed with the jMRUI version 5.0 software package. The raw datasets were Hamming-filtered in *k*-space, 2D Fourier transformed, and residual water was removed in the time domain with an HLSVD filter^1^. To obtain the peak areas and chemical shifts of ISUCA from the spectra, the ISUCA peaks were fitted to either a Lorentzian or Gaussian line shape using the time-domain AMARES algorithm^2^, with prior knowledge of peak constraints. The unsuppressed water MRSI dataset was used to reference the ISUCA resonance chemical shifts in each voxel to water at 4.7ppm.

A Python program was written to construct pH_e_ maps from the spectral datasets. This included quality control constraints to discard voxels of poor spectral quality and those outside the PRESS-localized ROI (and therefore outside the tumour). Using the pH calibration curve for ISUCA, the pH_e_ values were calculated from the water frequency-corrected ISUCA H2 chemical shifts and exported both as a false-colour pH_e_ map and as a spreadsheet. Any voxel found to contain underlying skeletal muscle on the T_2_-weighted image was subsequently excluded from the pH_e_ map. The pH values in this final pH_e_ map were used for statistical analyses.

For each voxel in the final pH_e_ map, the concentration of ISUCA was measured by calculating the peak area of the ISUCA H2 peak normalized to the unsuppressed water peak and by using the assumption that tissue water proton concentration was 88M^3^. The concentrations of ISUCA obtained from the individual voxels that passed the quality control tests were used for statistical analysis of the correlation between pH and ISUCA concentration to test whether the buffering effect of ISUCA was sufficient to bias the results.

**^31^P Single-Voxel Data Processing**

Single-voxel data were processed in jMRUI version 5.0. Gaussian peaks were fitted to phosphomonoester (PME), inorganic phosphate (P_i_), phosphodiester (PDE), phosphocreatine (PCr), γ-adenosine triphosphate (γ-ATP), α-ATP, β-ATP and reduced nicotinamide adenine dinucleotide (NADH), by imposing prior knowledge of peak frequencies. pH_i_ was calculated from the chemical shift between P_i_ and α-ATP; the PCr signal was not used, as this is typically very low in tumour tissue and any detected may be contamination from muscle underlying the tumour. Only tumours with signal to noise ratio (SNR) > 5 were accepted for analysis. The SNR was calculated from the jMRUI results as the ratio of amplitude to standard deviation for the α-ATP resonance.

**Analysis of Lactate in Tumour** **Samples**

Tumours (CA9 constitutive expressers and EV5 empty vectors) were excised from terminally anesthetized mice and immediately freeze-clamped using tongs previously immersed in liquid nitrogen. Samples were powdered in liquid nitrogen and extracted with 4 volumes of 6% perchloric acid followed by centrifugation and neutralization. Lactate was assayed in the extracts using the L-Lactate Assay Kit (Colorimetric) ab65331 (Abcam) according to the manufacturer’s instructions. Since lactate is a passively distributed solute, total tissue measurements cannot attribute its location relative to the cell membrane. The pH distribution was therefore used to infer the intracellular to extracellular lactate gradient.

**References**

1. Van den Boogart A, Van Ormondt D, Pijnappel W, De Beer R, Ala-Korpela M *Mathematics in Signal Processing III*. Oxford: Clarendon Press; 1994.
2. Vanhamme L, van den Boogaart A, Van Huffel S. (1997) Improved method for accurate and efficient quantification of MRS data with use of prior knowledge. *J Magn Reson* **129**: 35-43.
3. Madhu B, Waterton JC, Griffiths JR, Ryan AJ, Robinson SP. (2006) The response of RIF-1 fibrosarcomas to the vascular-disrupting agent ZD6126 assessed by *in vivo* and *ex vivo* ^1^H magnetic resonance spectroscopy. *Neoplasia* **8**: 560-567.
